# Application of metagenomic next-generation sequencing in the diagnosis of pathogens in patients with diabetes complicated by community-acquired pneumonia

**DOI:** 10.1515/biol-2022-1048

**Published:** 2025-03-18

**Authors:** Hong-bo Chen, Jie Liu, Yu Zhang, Hao Huang, Li-na Wang

**Affiliations:** Department Respiratory Medicine, Anning First People’s Hospital Affiliated to Kunming University of Science and Technology, No. 2 of South Gang He Road, Anning, Kunming, 650302, Yunnan, China; Department Medical Records Statistics Section, Anning First People’s Hospital Affiliated to Kunming University of Science and Technology, No. 2 of South Gang He Road, Anning, Kunming, 650302, Yunnan, China

**Keywords:** bronchoalveolar lavage fluid, community-acquired pneumonia, diabetes, metagenomic next-generation sequencing, pathogenics

## Abstract

To explore the clinical utility and optimal timing of metagenomic next-generation sequencing (mNGS) in diagnosing pathogens in patients with diabetes complicated by community-acquired pneumonia (CAP). The study included 50 hospitalized patients diagnosed with diabetes complicated by CAP who underwent conventional microbiological testing (CMT) and mNGS using bronchoalveolar lavage fluid. Among the 50 cases, 16% presented no respiratory symptoms. There were significant increases in inflammatory markers such as C-reactive protein, erythrocyte sedimentation rate, and interleukin-6, with patchy imaging changes being the most prevalent. The positive rates for pathogen detection by mNGS and CMTs were 78 and 21% (*P* < 0.05). The mNGS was significantly better than the CMTs in the detection of rare pathogens such as *Anaerobes*, *Chlamydia psittaci, Legionella pneumophila, Mycobacterium bovis, Aspergillus fumigatus*, and *Pneumocystis japonicus* (*P* < 0.05). After clinical interpretation, 85% (22/26) of viruses, 24% (9/37) of bacteria, and 25% (2/8) of fungi were non-pathogen organisms by mNGS. There was a significant difference in the rates of adjustment in anti-infection treatment strategies based on the pathogen detection results from CMTs and mNGS, which were 2 and 46%, respectively (*P* < 0.05). We found that mNGS was superior to CMTs in terms of the positive rate of pathogen detection, detecting mixed infection incidence, rare pathogen detection rates, and the adjustment of treatment strategies. However, mNGS results need to be interpreted in the context of the clinic.

## Graphical abstract

The use of mNGS to detect pathogens in diabetes mellitus combined with community-acquired pneumonia is described, which can be more advantageous in terms of the positive rate of pathogen detection, mixed infections, and detection of rare pathogens, and can lead to timely adjustments in therapeutic strategies; however, mNGS results need to be interpreted in conjunction with the clinic.

**Figure:**
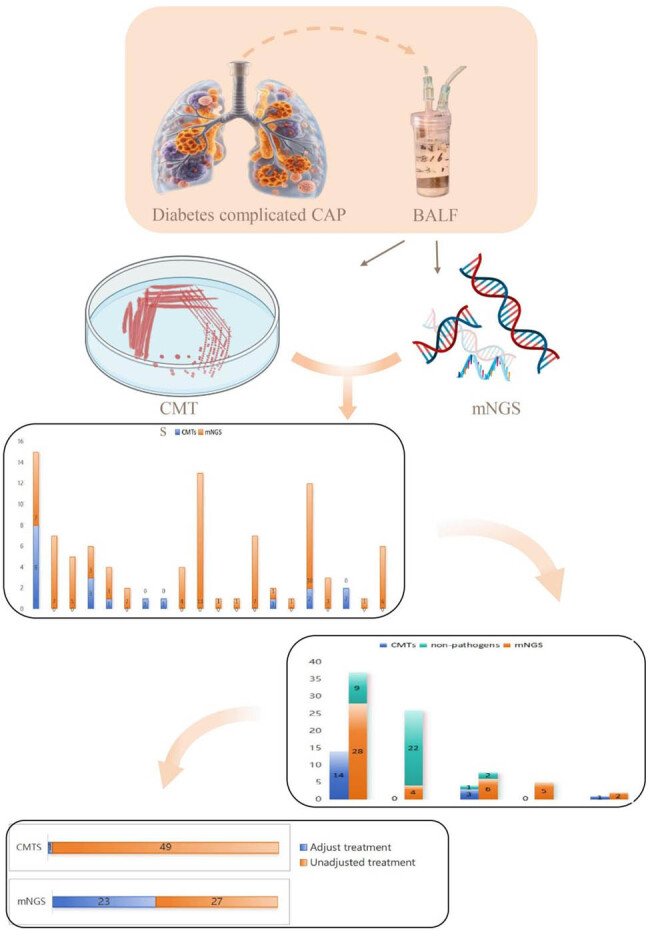

## 1 Introduction

Pulmonary infections are twice as likely in patients with diabetes compared to the healthy population [1]. The incidence of viral infections, pulmonary tuberculosis, fungal infections, and other opportunistic infections is higher in these patients compared to the general population due to a certain degree of immunosuppression and metabolic disorder [2]. Therefore, it is crucial to proactively use pathogenic diagnostics to selectively choose antimicrobial drugs that can quickly alleviate symptoms and reduce healthcare burdens.

Conventional microbiological testing (CMT) methods mainly include throat swab cultures, blood cultures, sputum and bronchoalveolar lavage fluid (BALF) smears and cultures, and serum pathogen-specific microbial antibody assays. However, CMTs often yield low positive rates, have insufficient coverage, and are time-consuming, thus falling short of meeting the clinical need for prompt and accurate assessments in such patients [3].

In recent years, metagenomic next-generation sequencing (mNGS) has been widely used in clinical settings. This technique sequences all nucleic acids in a sample, employing bioinformatics analysis to identify genes associated with pathogenic microorganisms, and has extensive coverage, as it is capable of detecting over 10,000 pathogens. An increasing number of clinical studies have validated the diagnostic efficacy of mNGS for infectious diseases [4–6].

However, the clinical utility of mNGS in patients with diabetes complicated by community-acquired pneumonia (CAP) remains inadequately explored. In this study, the clinical characteristics and pathogen spectrum of 50 patients diagnosed with diabetes complicated by CAP who were hospitalized between June 2021 and June 2023 were analyzed. The differences in pathogen detection results between BALF mNGS and CMTs were compared. This study aimed to investigate the use of mNGS in patients with diabetes combined with CAP.

## 2 Materials and methods

### 2.1 Study participants

Fifty patients treated in the respiratory intensive care unit of our hospital from June 2021 to June 2023 were selected for the study. The inclusion criteria were based on the Chinese adult CAP diagnostic criteria [7]. The exclusion criteria were as follows: (1) patients who could not tolerate bronchoscopy, (2) patients admitted for treatment of pulmonary infection within the last month, and (3) patients who were administered antimicrobial drugs for more than 1 week.


**Informed consent:** Informed consent has been obtained from all individuals included in this study.
**Ethical approval:** The research related to human use has been complied with all the relevant national regulations, institutional policies and in accordance with the tenets of the Helsinki Declaration, and has been approved by the Ethics Committee of the Anning First People’s Hospital.

### 2.2 BALF extraction and specimen submission

All patients underwent electronic bronchoscopy within 24–48 h of admission to the hospital. Based on imaging findings, lavage was performed two to three times in the affected lung segment or lobe, with 20 mL of fluid used for each lavage. The fluid was then reaspirated, divided into two portions, and placed in two sterile tubes, each containing at least 5 mL of BALF. The first tube was sent for routine pathogen testing, including smears for bacteria, fungi, and acid-fast bacillus, and cultures for bacteria and fungi. Library Construction and Metagenomic Sequencing DNA libraries were prepared using an automated nucleic acid extraction and library preparation instrument (Matridx, MA002). The workflow included nucleic acid extraction, enzymatic fragmentation, end-repair with A-tailing, adapter ligation, and purification [8]. Libraries were quantified using fluorescent quantitative PCR (KAPA), pooled in equimolar amounts, and subjected to high-throughput sequencing. Sequencing was performed on the Illumina NextSeq 550Dx platform, with an average data output of 20 million reads per library, read length of 75 bp, and dual-barcode sequencing. Each sequencing chip included one negative control (10⁴ Jurkat cells per mL of culture medium) and one positive control (a mixture of synthetic DNA or RNA fragments of adenovirus and influenza A virus, as well as inactivated bacterial, fungal, and pseudoviral particles) for quality control. Bioinformatics analysis of the raw sequencing data involved the following steps: removal of unnecessary adapter sequences and low-quality bases (library concentration >50 pM, Q20 > 85%, Q30 > 80%). Alignment of reads to the human reference genome (GRCh38.p13) using Burrows-Wheeler Alignment (BWA) to eliminate human host sequences. After removing low-complexity reads, the remaining sequencing data were aligned to reference databases (NCBI nt database and GenBank [9]) using BWA to identify microbial species [10].

### 2.3 Interpretation of positive test results

#### 2.3.1 Positive results in routine pathogen testing

If acid-fast bacilli were found in the BALF smear under microscopy, or if bacteria and/or fungi were cultured, the BALF routine pathogen testing results were considered positive.

#### 2.3.2 Interpretation of positive mNGS test results

(1) Considering the variation in microbial genome sizes, the detected reads were normalized based on the number of reads per million sequences (RPM). Positive detection thresholds were determined according to the type of pathogen. (2) A negative control (NC) in the same sequencing run is defined as containing no detectable species or having an RPM(sample)/RPM(NC) ratio of ≥5, which has been empirically established as the cutoff to distinguish true positives from background contamination [11]. (3) For conditionally pathogenic bacteria associated with CAP and hospital-acquired pneumonia, those with a relative abundance >30% at the genus level were included in the list of pathogens of interest [12]. In the lower respiratory tract, low-pathogenicity or colonizing microorganisms were considered potentially pathogenic only if their relative abundance was ≥50% [13]. Hard-to-lyse microorganisms, such as the *Mycobacterium tuberculosis* complex, were identified based on at least one specific sequence read [14]. Diagnosis of tuberculosis infection further required supporting clinical evidence of tuberculosis positivity. (4) Fungi: an RPM ≥5 was considered a positive threshold, with greater reliability achieved as the sequencing read count increased. Parasites: an RPM ≥10 was considered positive, provided that the mapped reads corresponded to specific sequences [15]. (5) Viruses: detection was reported if the reads covered at least three non-overlapping regions of the viral reference genome, each ≥140 bp in length [16]. (6) Environmental or laboratory contamination, reagent contamination, or commensal microbiota such as human skin flora and normal microbiota (e.g., papillomavirus) detected in no template control and positive control samples were excluded from reporting.

#### 2.3.3 Concordance rate between mNGS and CMTs

The concordance rate between mNGS and CMTs was determined by comparing the pathogens detected by both methods. If the same pathogens were detected in the positive results of both tests, they were considered concordant.

### 2.4 Formulating a clinical treatment strategy

To formulate an effective clinical strategy, the clinical presentation, laboratory tests, imaging studies, and results from mNGS and CMTs of the patient were collected and comprehensively analyzed by a multidisciplinary team consisting of respiratory physicians, microbiology laboratory physicians, and radiologists. Considering the patient’s history, immune status, clinical presentation, and chest imaging features, positive results were repeatedly evaluated by this team to determine the presence of infection, and the anti-infection treatment strategy was adjusted accordingly.

### 2.5 Statistical analysis

Statistical methods: Data analysis was performed using SPSS 23.0 statistical software. Quantitative data conforming to normal distribution were expressed as mean ± standard deviation ( \[\bar{x}]\] ± \[s]\] ), while other data were expressed as the median (P_75_, P_25_). Group comparisons were conducted using the independent samples *t*-test for normally distributed data and the Mann–Whitney *U* test for non-normally distributed data. Count data were compared using the *χ* ^2^ test, with a significance level set at a *P* value of <0.05. When the expected frequency in any cell of a 2 × 2 table was <1, the Fisher’s exact test was used.

## 3 Results

### 3.1 Clinical characteristics of patients

Clinical details that were collected from 50 patients with diabetes and CAP included their age, gender, body temperature, white blood cell count, absolute neutrophil count, serum C-reactive protein (CRP), procalcitonin (PCT), erythrocyte sedimentation rate (ESR), interleukin-6 (IL-6), CD4^+^, CD8^+^, CD3^+^, NK cells, imaging changes, duration of hospitalization, number of improved cases, and number of deaths. Details are shown in Table 1. Among them, 20% of the patients (10/50 cases) had immune dysfunction with CD4^+^ T lymphocyte counts below 200 cells/µL.

**Table 1:** Overview of clinical data of patients with diabetes and CAP

Item	Number of cases (*n* = 50)	Item	Number of cases (*n* = 50)
Age (years, mean ± SD)	64.74 ± 14.65	CD8^+^ (cells/µL, *M* (*Q*_1_, *Q*_3_))	243.10 (132.16, 410.46)
Male (number of cases [%])	32 (64%)	CD3^+^ (cells/µL, *x̅* ± *s*)	577.86 ± 399.50
Body temperature (°C, *M*(*Q*_1_, *Q*_3_))	36.60 (36.33, 37.48)	NK cells (%, *x̅* ± *s*)	11.37 ± 9.94
White blood cell count (10^9^/L, *M*(*Q*_1_, *Q*_3_))	8.93 (8.17, 13.11)	Consolidation in both lungs (number of cases [%])	8 (16.00%)
Absolute neutrophil count (10^9^/L, *M*(*Q*_1_, *Q*_3_))	6.85 (4.39,9.98)	Patchy shadow (number of cases [%])	33 (66.00%)
Serum CRP (mg/L, *x̅* ± *s*)	94.54 ± 82.76	Ground glass opacity (number of cases [%])	9 (18.00%)
Serum PCT (ng/L, *M*(*Q*_1_, *Q*_3_))	0.15 (0.07, 0.70)	Respiratory symptoms (number of cases [%])	42 (84.00%)
ESR (mm/h, *x̅* ± *s*)	52.25 ± 38.31	Hospitalization duration (*d*)	10.20 ± 6.77
IL-6 (mg/L, *M*(*Q*_1_, *Q*_3_))	22.69 (10.34, 76.41)	Improved (number of cases [%])	48 (96.00%)
CD4^+^ (cells/µL, *x̅* ± *s*)	416.87 ± 265.63	Death (number of cases [%])	2 (4.00%)

### 3.2 Comparison of pathogen detection by CMTs and mNGS in patients with diabetes and CAP

**Figure 1:**
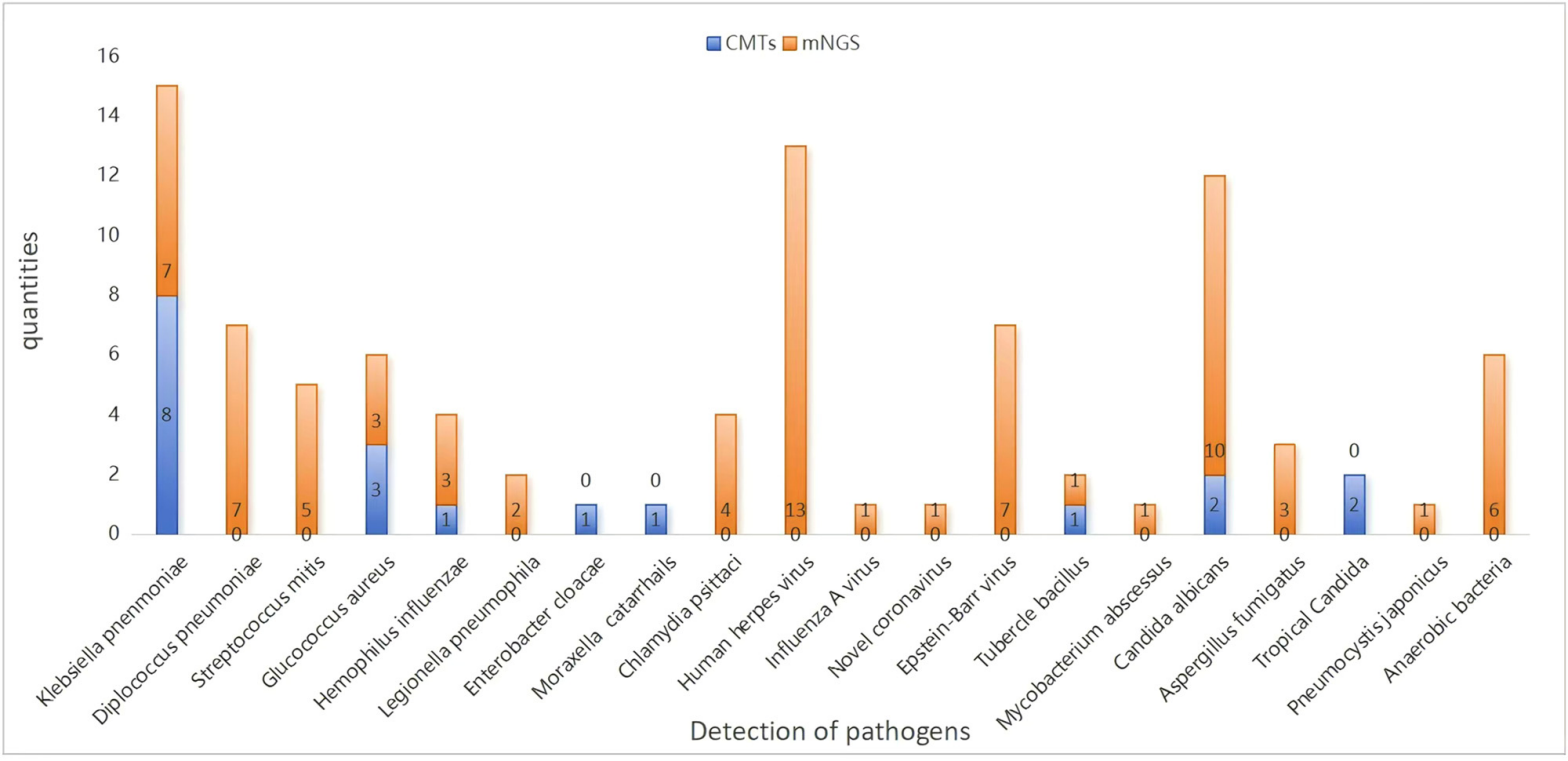
Pathogens detected by CMTs and mNGS in patients with diabetes combined with CAP.

The composition of pathogens detected by CMTs and mNGS in patients with diabetes combined with CAP is shown in Figure 1, from which it can be seen that mNGs have a significant advantage over CMTs in terms of the range of detection, detection of *Anaerobes, Streptococcus pneumoniae, Streptococcus bradypneumoniae*, viruses, atypical pathogens*, Aspergillus*, *Pneumocystis japonicus*, *Legionella*, and *Mycobacterium*.

**Figure 2:**
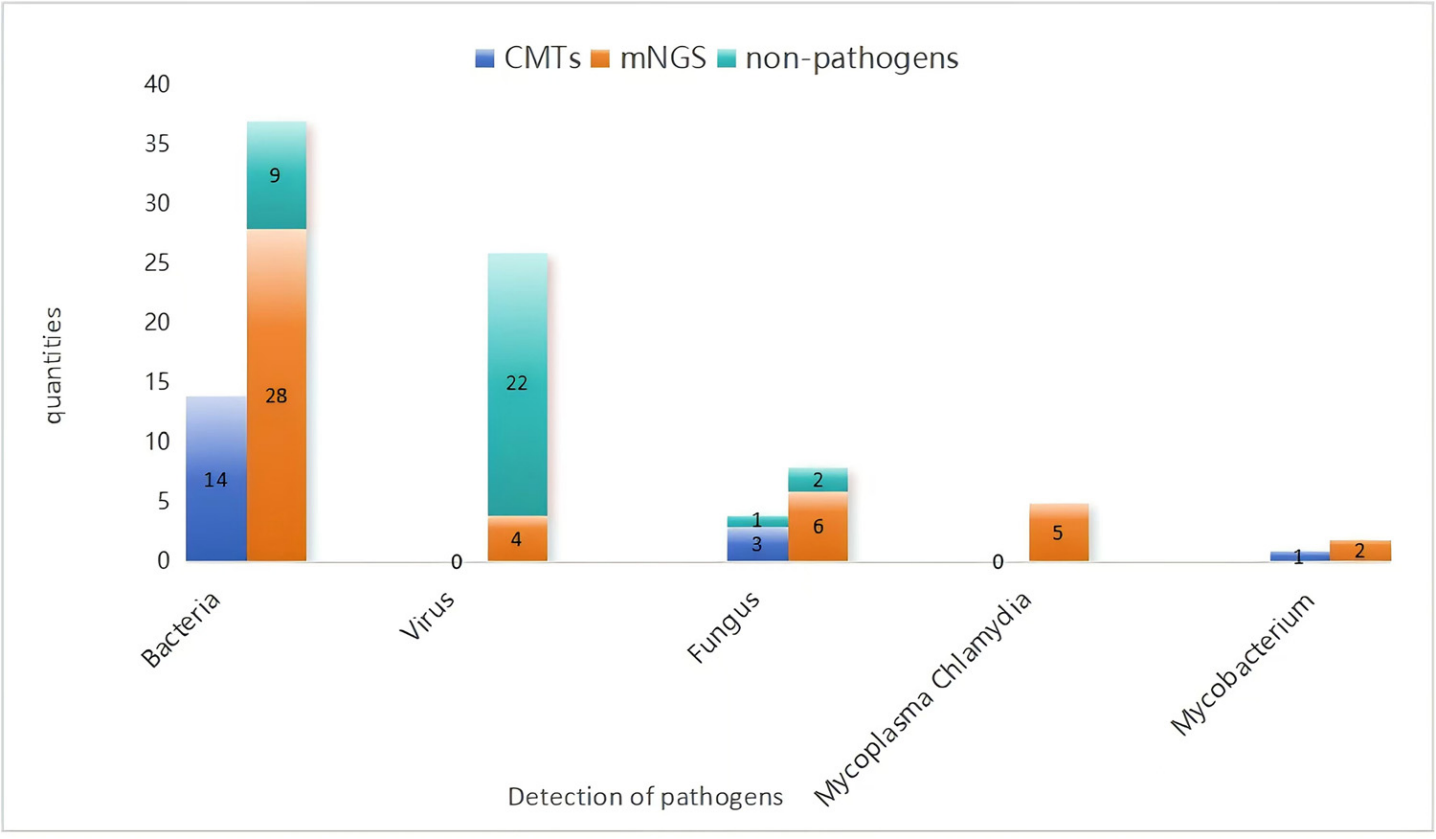
Non-pathogens after discriminating detected by CMTs and mNGS in patients with diabetes combined with CAP.

Pathogens detected in the ten immunocompromised patients included three cases of *Chlamydia psittaci*, three cases of *Klebsiella pneumoniae*, two cases of *Staphylococcus aureus*, one case of *Legionella pneumophila*, and one case of *Aspergillus fumigatus*. CMTs and mNGS in combination with the patient’s clinical presentation, laboratory tests, and imaging studies were investigated as non-pathogen organisms and are shown in Figure 2, in which 85% (22/26) of viruses, 24% (9/37) of bacteria, and 25% (2/8) of fungi were non-infectious organisms in the results of mNGS.

### 3.3 Comparison of diagnostic performance between CMTs and mNGS in patients with diabetes and CAP

In the 50 cases of diabetes complicated with CAP, the positive rates of pathogen detection by mNGS and CMTs in BALF were 78 (39/50 cases) and 21% (12/50 cases), respectively. Comparisons between mNGS and CMTs showed statistically significant differences in the positive rate of pathogen detection and the detection of mixed infections (*P* < 0.05). mNGS was significantly superior to CMTs in detecting rare pathogens such as *anaerobic bacteria*, *C. psittaci*, *L. pneumophila*, *Mycobacterium* species, *A. fumigatus*, and *Pneumocystis jiroveci* (*P* < 0.05) (Table 2), with *anaerobic bacteria* detected in six cases and *C. psittaci* detected in four cases.

**Table 2:** Comparison of pathogen detection positive rates between mNGS and CMTs among all patients

Group		Positive rate	Single infection	Mixed infection	Rare pathogen infection
mNGS	50	39 (78.0%)	20 (40.0%)	19 (38.0%)	11 (22.0%)
CMTs	50	12 (24.0%)	12 (24.0%)	0 (0.0%)	0 (0%)
*χ* ^2^ value		29.172	2.941	23.457	12.360
*P* value		0.000	0.085	0.000	0.000

### 3.4 Concordance between mNGS and CMTs methods

The consistency of the mNGS method with the CMTs method was mainly in bacteria and Candida. For the detection of bacteria, the bacterial species with the highest abundance detected by mNGS matched the bacterial culture results from CMTs in six instances. However, there were four cases where the bacterial results detected by CMTs did not match the most abundant bacteria detected by mNGS. Specifically, CMTs detected *Escherichia coli* and *K. pneumoniae*, whereas the most abundant bacteria detected by mNGS was *Streptococcus mitis*. For fungi, there were three specimens in which *Candida albicans* detected by the mNGS method was consistent with *C. albicans* from the fungal culture results of the CMTs method.

### 3.5 Changes in antimicrobial drugs

Subsequent to a comprehensive analysis by respiratory physicians, microbiology laboratory physicians, and radiologists, positive results were utilized to confirm infections, and anti-infection treatment strategies were adjusted based on the results from CMTs and mNGS. The adjustment rates were 2% (1/50 cases) in the case of CMTs and 46% (23/50 cases) in mNGS, with a statistically significant difference between the two methods (*P* < 0.05). The main changes that were made included switching to targeted treatment with quinolones, tetracyclines, vancomycin, triazole antifungals, macrolides, 3CL protease inhibitors, and antituberculosis drugs.

## 4 Discussion

The prevalence rate of adult diabetes in China is 11.2%, [17] with a high prevalence of diabetes as complication. In this study, the clinical characteristics of 50 cases of diabetes complicated with CAP were examined. There were no respiratory symptoms in 16% of patients, and increases in body temperature, white blood cell count, and absolute neutrophil count were uncommon. However, inflammatory markers, such as CRP, ESR, and IL-6, were significantly elevated. The most common imaging changes were patchy imaging changes. Significant abnormalities in the CD4^+^ T lymphocyte count and CD4^+^/CD8^+^ ratio in patients with diabetes combined with pulmonary infection have been reported in the literature [18]. In this study, we found that the average CD4^+^ T lymphocyte count was >400 cells/µL, with only ten cases having counts <200 cells/µL, suggesting that not all patients with diabetes exhibit immune dysfunction.

Over half of the patients with pulmonary infectious diseases remain undiagnosed with pathogens when traditional laboratory examinations are used. The use of mNGS has significantly improved the pathogen detection rate, thereby enhancing the understanding of pathogens involved in diabetes complicated by CAP. The bacterial pathogens detected in this study are consistent with those reported earlier [19]. Compared to standard CAP, the chances of infection with special pathogens like *Mycobacterium*, *Aspergillus*, and *Legionella* are significantly higher [19]. In this study, pathogens such as *Anaerobic bacteria*, *C. psittaci*, *L. pneumophila*, *A. fumigatus*, and *P. jiroveci* were detected.

Notably, diabetes patients complicated with CAP and in an immunocompromised state are more prone to infections by rare pathogens such as *S. aureus* and pulmonary *Aspergillus* [20]. In this study, *C. psittaci* was detected in three cases, *K. pneumoniae* in three, *S. aureus* in two, *L. pneumophila* in one, and *A. fumigatus* in one – all these were identified through mNGS. This suggests that for patients with diabetes and CAP who have cellular immune deficiencies, the use of mNGS can enhance pathogen detection.

Results in BALF routine bacterial and fungal cultures typically require 3–5 days, whereas mNGS testing can be completed within 24–36 h. Therefore, mNGS, with its high sensitivity for pathogen detection and shorter testing duration, has gradually emerged as one of the most promising molecular diagnostic techniques for pathogen detection [21], but mNGS costs almost ten times more than CMTs. In this study, the positive rate for pathogen detection by mNGS was 78%, which was significantly higher than the CMTs detection rate of 24%. This finding was consistent with the literature [22]. mNGS is superior to CMTs in detecting bacteria, viruses, and mycoplasma. When mNGS and CMTs were compared, there was also a significant difference in the detection of mixed infections and rare pathogens (*P* < 0.05). mNGS clearly outperformed CMTs in detecting viruses, including *C. psittaci*, *L. pneumophila*, *Mycobacterium abscessus*, *A. fumigatus*, *Anaerobic bacteria*, and *P. jiroveci*. Furthermore, based on mNGS results, 46% of patients (23/50 cases) had their antimicrobial drugs adjusted, deliberately reducing the need for empirical antimicrobial treatment that covers all pathogens.

mNGS exists in a wide range of assays and the results require clinical screening. In this study, using comprehensive analyses, the treating team consisting of respiratory physicians, microbiology laboratory physicians, and radiologists identified that, even with low sequence counts, it was necessary to consider pathogens such as *Legionella*, *C. psittaci*, *Mycoplasma pneumoniae*, and *Mycobacterium tuberculosis* as pathogenic, whereas *Candida*, *Aspergillus*, and *P. jiroveci* could be colonizing organisms rather than pathogens. mNGS has a significant advantage in virus detection, but attention should only be paid to pathogenic viruses such as COVID-19, *cytomegalovirus*, *Adenovirus*, and *influenza* viruses [23].

Although patients with diabetes complicated by CAP carry risks of opportunistic pathogen infections and drug-resistant bacteria, it is still feasible to initiate empirical treatment after a thorough evaluation of the patient’s condition. mNGS shows significant advantages in detecting pathogens with high positive rates, mixed infections, and rare pathogens, thereby facilitating the timely adjustment of treatment strategies. However, mNGS results need to be interpreted in the context of the clinic. In cases of immune dysfunction, it is recommended that samples be immediately sent for mNGS testing to benefit patient recovery. There were several limitations. First, the current study only included a small number of cases. Second, the sample in this study predominantly reflects a specific ethnic group, which may limit the generalizability of our findings to other ethnicities. Future studies should aim to include a more ethnically diverse cohort with larger sample size to better understand the role of genetic variations in disease susceptibility and pathogen identification.
